# Characterization and Potential Antitumor Activity of Polysaccharide from *Gracilariopsis lemaneiformis*

**DOI:** 10.3390/md15040100

**Published:** 2017-03-29

**Authors:** Yani Kang, Zhi-Jiang Wang, Dongsheng Xie, Xue Sun, Wenge Yang, Xiaodong Zhao, Nianjun Xu

**Affiliations:** 1 Key Laboratory of Marine Biotechnology of Zhejiang Province, School of Marine Sciences, Ningbo University, Ningbo 315211, Zhejiang, China; kangyani@sjtu.edu.cn (Y.K.); sunxue@nbu.edu.cn (X.S.); yangwenge@nbu.edu.cn (W.Y.); 2 School of Biomedical Engineering, Bio-ID Research Center, Shanghai Jiao Tong University, Shanghai 200240, China; xiaodongzhao@sjtu.edu.cn; 3 College of Biological and Environmental Sciences, Zhejiang Wanli University, Ningbo 315100, China; wangzj1978@126.com; 4 School of Pharmacy, Shanghai Jiao Tong University, Shanghai 200240, China; dshxie@sjtu.edu.cn

**Keywords:** polysaccharide, *Gracilariopsis lemaneiformis*, antitumor activity, Fas/FasL pathway, cell proliferation, apoptosis

## Abstract

Substances with valuable antitumor properties have been identified in many marine algae, including an edible polysaccharide from the marine alga *Gracilariopsis lemaneiformis* (PGL). We previously reported transcriptome profiling data showing that PGL induced transcriptional alterations generate anti-lung cancer activity. To identify how PGL is detrimental to tumors, we purified PGL to characterize its chemical composition, molecular weight, and sugar and protein content and investigated its antitumor activity. We demonstrated that PGL exerted its antitumor activities by modulating cell viability, morphology, apoptosis, and the apoptosis-related Fas/FasL signaling pathway in the human lung cancer cell line A549, the gastric cancer cell line MKN28, and the mouse melanoma cell line B16. Our data provide the first evidence that PGL inhibits cell proliferation by inducing apoptosis, which is largely mediated by Fas/FasL in cancer cells, suggesting that PGL might be a novel therapeutic agent against cancer.

## 1. Introduction

Cancer is a serious medical challenge requiring a proper therapeutic approach with fewer side effects. Recently, marine algae have been explored for potential anticancer agents, although the use of polysaccharides as antitumor therapies is under intense debate [1,2]. Many of the polysaccharides found in marine creatures have been assessed for their anticancer properties, and some have been widely investigated in vitro and in vivo [3,4]. As natural bioactive compounds, antitumor products such as algal polysaccharides are used widely in pharmaceuticals as anticancer agents [5,6]. The marine macroalga *Gracilariopsis lemaneiformis* (*Gp. lemaneiformis*) is an important, commercially valuable, renewable biomass resource in the marine environment with beneficial health effects [7]. Polysaccharides are the key active antitumor substances obtained from *Gp. lemaneiformis* [8], and their structure and activity are the basis for medicinal and health care applications [1,9,10]. Polysaccharides from *Gp. lemaneiformis* (PGL) consist of 3,6-anhydro-l-galactose and d-galactose and are acidic polysaccharides with a linear structure of repeated disaccharide agarobiose units [11]. Since polysaccharide bioactivity is most closely related to their chemical composition, configuration, and molecular weight (MW), as well as their physical properties, we extracted and purified PGL using chromatography and partially characterized it using a series of chemical and instrumental analyses. In addition, its antitumor activities were analyzed in vitro.

We previously showed that PGL significantly inhibits lung cancer cell proliferation and changes cell morphology [12]. Moreover, our transcriptome analysis demonstrated that PGL induced lung cancer apoptosis and cell cycle arrest by modulating the expression of related genes [13]. In this study, we further investigated PGL antitumor activity in the human gastric cancer cell line MKN28, the lung cancer cell line A549, and the mouse melanoma cell line B16 using CCK-8 assays, phase-contrast microscopy, annexin V-FITC/PI staining, flow cytometry, RT-qPCR, western blotting, and transfections.

The Fas/Fas ligand (Fas/FasL) pathway plays a significant role in tumorigenesis, and its impairment in cancer cells leads to apoptotic resistance and contributes to tumor progression [14,15]. Emerging evidence suggests that Fas ligand activation enhances Fas-dependent apoptosis and induces robust immune responses against tumors [2]. Since Fas/FasL signaling plays a vital role in regulating apoptosis, we investigated whether PGL-treated cells induced Fas and FasL expression. This is the first study showing that PGL exerts its antitumor effects by altering the Fas/FasL system. We demonstrated that PGL inhibits cancer cell proliferation by inducing apoptosis, which is largely mediated by the Fas/FasL system. Our results provide new insight into the mechanism of PGL’s antitumor properties.

## 2. Results and Discussion

### 2.1. Characterization of Polysaccharides from Gp. lemaneiformis

**Figure 1:**
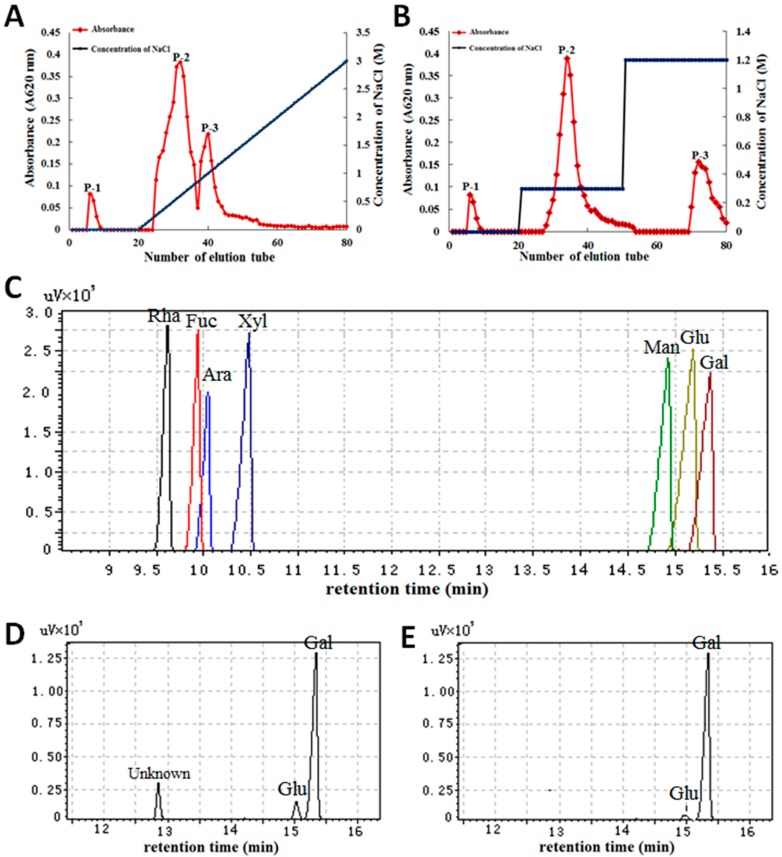
The purification and composition analysis of the polysaccharides from *Gp. lemaneiformis.* (**A**) Elution profiles of crude PGL on a DEAE-Sephadex A-25 ion exchange column; (**B**) PGL elution curve of polysaccharide fractions further purified on a Sephadex G-100 column equilibrated with distilled water; (**C**) Gas chromatogram of the monosaccharide standards; (**D**) Monosaccharide composition of the P-2 fraction; (**E**) Monosaccharide composition of the P-3 fraction.

It is critical to identify and extract the valuable and safe polysaccharides from *Gp. lemaneiformis* for medicinal applications. In this study, crude polysaccharides were extracted from the macroalga *Gp. lemaneiformis* and purified first by DEAE-A25 cellulose chromatography and then by Sephadex G-100 size-exclusion chromatography. The polysaccharide content was 93.57% from the crude polysaccharides (Table 1), and three main fractions were obtained from the purification steps, with each fraction generating a single elution peak called P-1, P-2, and P-3 (Figure 1A,B). Each fraction had only one main peak, and the main peaks were collected, dialyzed, desalted, concentrated, and lyophilized for use in subsequent assays.

The content and MW of these polysaccharides were different. The monosaccharide composition of the main fractions (P-2 and P-3) was determined by gas chromatography-mass spectrometry (GC-MS). The standard monosaccharides from left to right in the order of sugar, alcohol, and acetate in the gas chromatogram were rhamnose (Rha), fucose (Fuc), arabinose (Ara), xylose (Xyl), mannose (Man), glucose (Glu), and galactose (Gal) (Figure 1C). The monosaccharide composition was an 11.68:1:2.16 molar ratio of galactose, glucose, and an unknown monosaccharide (based on the area under the peak for each monosaccharide) (Figure 1D). P-3 exhibited a 1:32.78 molar ratio of glucose to galactose (Figure 1E). An analysis of the monosaccharide constituents revealed that the primary monosaccharide composition of purified PGL contained d-galactose and 3,6-anhydro-l-galactose, which is identical to a previous similar study on the monosaccharide composition of PGL using gel chromatography and chemical analyses [16].

Furthermore, the chemical composition, molecular weight and ultraviolet (UV) spectrum of the three fractions were analyzed. The purified products P-1, P-2, and P-3 were confirmed as polyacrylamide gel electrophoresis (PAGE) bands and single elution peaks with yields of 12.61%, 69.26%, and 18.70%, respectively. The average MW of PGL and the P-1, P-2, and P-3 fractions were 123.06, 14.29, 64.78, and 57.02 kDa, respectively (Table 1). Compared with PGL, the sulfate radical content in the main fractions of PGL did not change significantly, which is favorable for bioactivity. UV spectroscopy was applied to determine the protein and nucleic acid content in the polysaccharide fractions. The GC-MS assay demonstrated that the fractions were polysaccharides. 

### 2.2. PGL Inhibits Cell Proliferation

**Figure 2:**
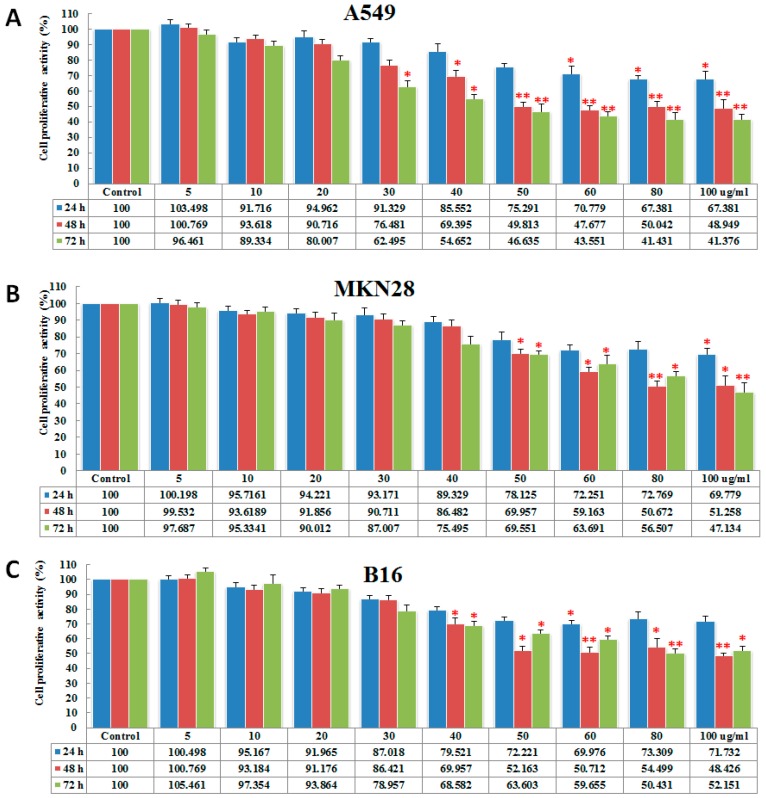
Inhibitory effects of serial PGL concentrations on proliferation in cancer cells. (**A**) The non-small cell lung cancer (NSCLC) cell line, A549; (**B**) The human gastric cancer cell line MKN28; (**C**) The mouse melanoma cell line B16. Three cancer cell lines were treated with PGL for 24, 48, and 72 h, and cell proliferation was examined using the CCK-8 assay. The columns are expressed as the mean ± SD of five samples from each treatment group. * *p* < 0.05 and ** *p* < 0.01 indicate significant differences between the control and PGL-treated groups. The data represent the results of five independent experiments.

The anticancer activity of polysaccharides has been reported frequently in recent years, and the potential mechanisms of action of polysaccharides were investigated [17]. In this study, we first examined the effect of PGL on cell proliferation. Three cancer cell lines (MKN28, A549, and B16) were treated with 0, 5, 10, 20, 30, 40, 50, 60, 80, or 100 μg/mL PGL for 24, 48, and 72 h. The cell proliferation of the PGL-treated cells was tested using a cell counting kit-8 (CCK-8) assay. The results showed that PGL does not inhibit cell proliferation at low concentrations (5, 10, and 20 μg/mL; the percentage of inhibition is less than 20% for all of the tested cell lines). When the concentration is more than 30 μg/mL, PGL inhibited cell growth in a dose- and time-dependent manner in A549, MKN28, and B16 cells, with the most robust effect in A549 cells (Figure 2A–C). The IC_50_ for PGL was approximately 50, 78, and 90 μg/mL after 48 h incubation in A549, MKN28, and B16 cells, respectively. These results are consistent with our previous report [13]. 

### 2.3. PGL Changes Cell Morphology and Reduces Cell Number

**Figure 3:**
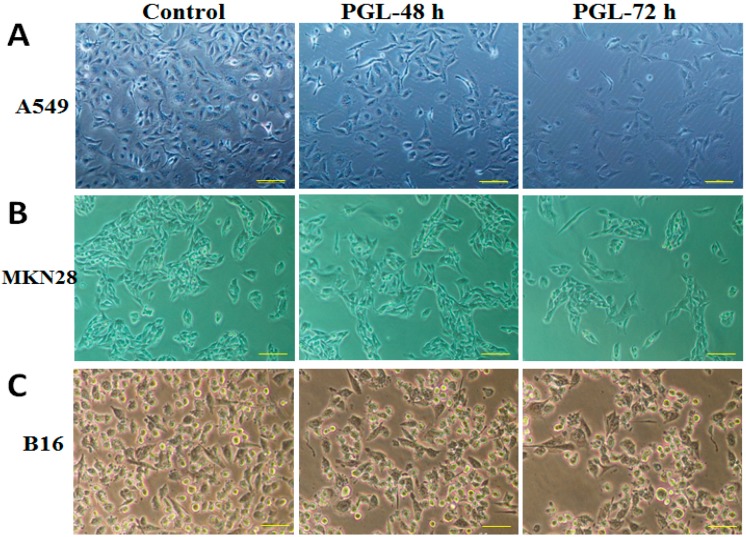
Effects of PGL on cell morphology in different cancer cells. Morphology changes were examined and photographed with phase-contrast microscopy in (**A**) the A549 human lung cancer cell line; (**B**) the MKN28 gastric cancer cell line; and (**C**) the B16 mouse melanoma cell line. The scale bar is 100 µm.

To examine the effect of PGL on morphology, changes in cell characteristics were examined and photographed using phase-contrast microscopy. As shown in Figure 3, the control cells exhibited intact nuclear membranes, dense growth, contact, and a normal morphology. Compared with the control, cells treated with PGL (60 µg/mL) for 48 h exhibited chromatin accumulation inside the nuclear membrane, a large number of autophagocytic vacuoles, and damaged mitochondria. After a 72 h of incubation with PGL (60 µg/mL), the cancer cells became smaller, organelles were destroyed, partial nuclear membranes were disrupted, and some nuclei even fragmented. With increasing time, the irregular changes in cell morphology, growth, and cell contacts decreased significantly in A549, MKN28, and B16 cells, confirming significant PGL antitumor activity (Figure 3A–C).

### 2.4. PGL Induces Apoptosis

**Figure 4:**
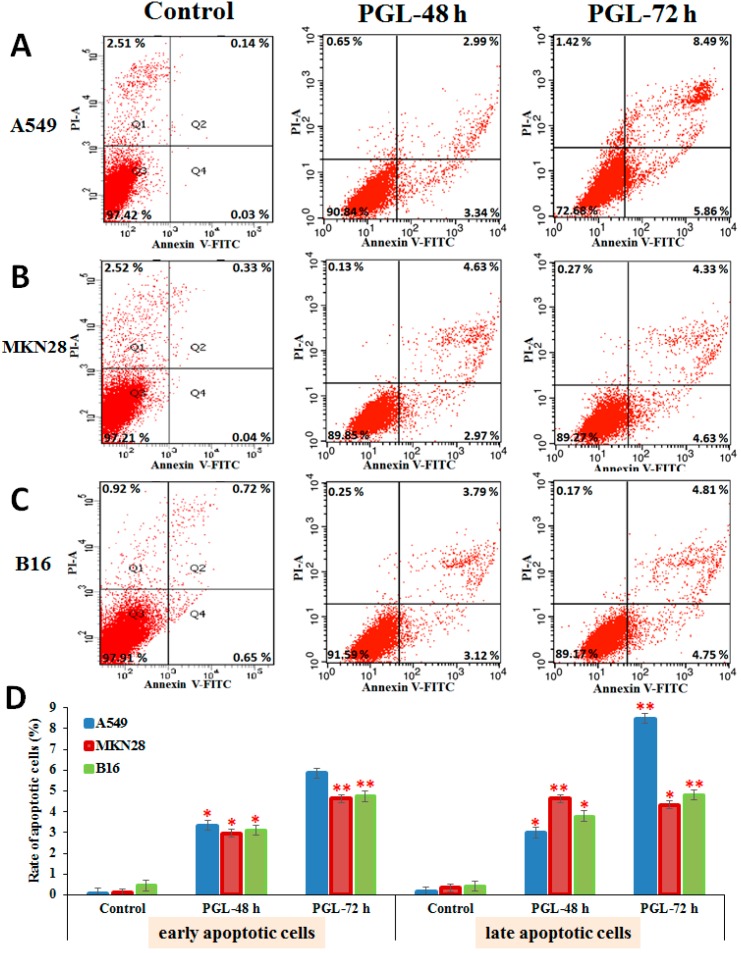
PGL induced apoptosis in A549, MKN28, and B16 cells. Apoptotic cells were detected by flow cytometry after PGL (60 µg/mL) treatment for 48 and 72 h in (**A**) the human lung cancer cell line A549; (**B**) the gastric cancer cell line MKN28; and (**C**) the mouse melanoma cell line B16; (**D**) Early and late apoptotic rates were analyzed by flow cytometry. Bars = the mean ± S.D., *n* = 3, * *p* < 0.05 and ** *p* < 0.01 indicate significant differences between the control and PGL-treated groups.

Many polysaccharides can possess either a direct inhibitory action on cancer cells and tumors or influence different stages of carcinogenesis and tumor development, recover the broken balance between proliferation and programmed cell death (apoptosis), and be used for cancer prophylactics [18]. Apoptotic changes have been implicated in the pathogenesis of many diseases, including various types of cancers [19]. In this study, apoptotic cells were stained with propidium iodide (PI) and analyzed by flow cytometry within one hour. PGL induced apoptosis in a time-dependent manner in A549 (Figure 4A), MKN28 (Figure 4B), and B16 (Figure 4C) cells, with the most robust effect on A549 cells. The results suggest that MKN28 cells treated with PGL (60 µg/mL) were in the early stages of apoptosis, whereas PGL-treated A549 cells had already completed apoptosis (Figure 4D). In addition, we found that there were many necrotic cells in the control A549 and MKN28 cells, whereas there were no necrotic cells in the PGL-treated cells. This result demonstrated that cancer cells proliferated too quickly in the control group in the absence of inhibitory drugs. 

### 2.5. PGL Activates Fas/FasL Expression

**Figure 5:**
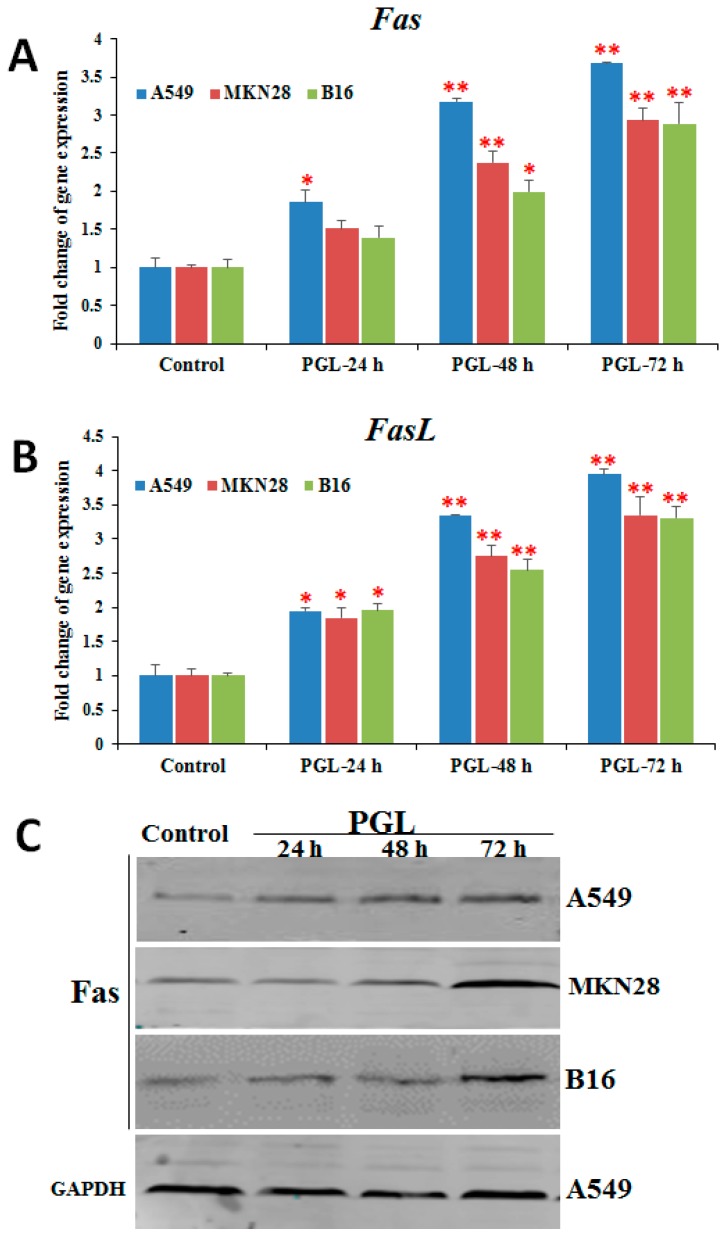
Effects of PGL on the expression of Fas and FasL in three cancer cell lines. (**A**) The mRNA expression of Fas after PGL treatment in three cancer cell lines. (**B**) The mRNA expression of FasL after PGL treatment in three cancer cell lines. (**C**) Fas protein levels in the three PGL-treated cancer cells, as assessed by western blotting. The three cancer cell lines were treated with 0 or 50 μg/mL PGL for 24, 48, and 72 h, and gene expression was examined by RT-qPCR. The columns show the mean ± SD of five samples for each group. * *p* < 0.05 and ** *p* < 0.01 indicate significant differences between the control and PGL-treated groups. The data are representative of five independent experiments.

The apoptotic death receptor pathway has previously been proposed as an anticancer drug target [2]. Fas, a member of the death receptor family, plays a vital role in apoptosis [20]. Many chemotherapeutic agents have been reported to induce Fas/FasL-mediated apoptosis in tumor cells through different mechanisms [15]. To explore the underlying molecular signaling pathways triggered by PGL, we examined the expression of Fas/FasL by RT-qPCR and western blotting. As shown in Figure 5, the expression of Fas and FasL was up-regulated in all three PGL-treated (60 µg/mL) cell lines at both the mRNA and protein levels (Figure 5). These results suggest that PGL induces apoptosis by activating genes involved in the death receptor apoptotic pathway and that the Fas/FasL signaling pathway might play a critical role in PGL-induced apoptosis.

### 2.6. Fas Knockdown Inhibits Cell Proliferation, Invasion, and Migration and Reduces Apoptosis

**Figure 6:**
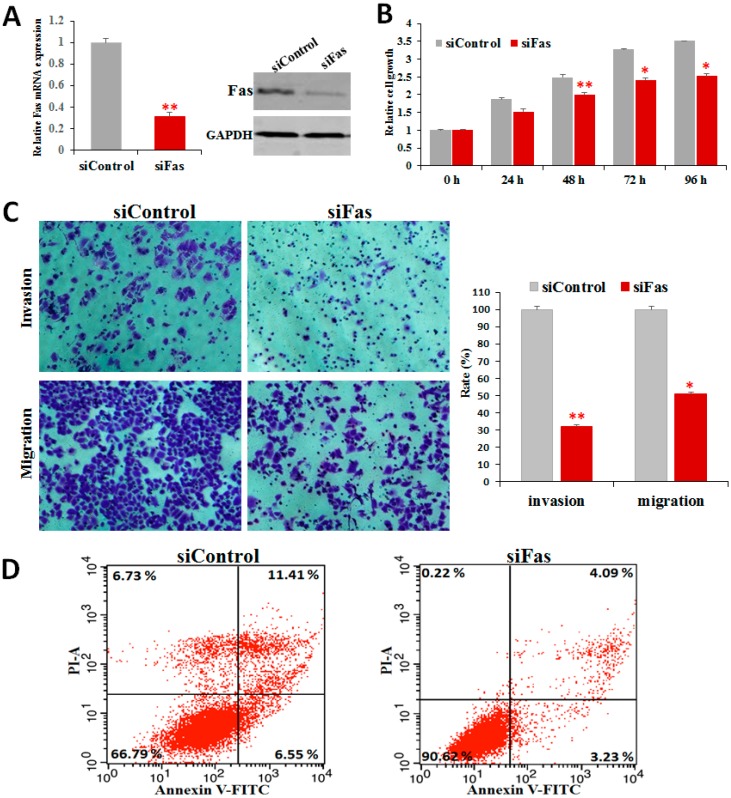
Downregulation of Fas inhibits cell proliferation, invasion, and migration and reduces apoptosis. (**A**) Fas expression decreased in cells after treatment with Fas-targeting siRNA (siFas) compared to control siRNA (siControl); (**B**) Fas knockdown inhibited lung cancer cell proliferation; (**C**) Fas knockdown suppressed lung cancer cell invasion and migration. (**D**) Fas knockdown reduced apoptosis in A549 lung cancer cells.

To determine whether Fas/FasL expression was critical for the antitumor effect of PGL, we knocked down Fas by siRNA and measured proliferation, invasion, migration, and apoptosis. Although PGL treatment increased Fas expression in all of the cell lines, we chose A549 cells for the detailed analysis because the Fas expression level in these cells was the highest. We successfully knocked down Fas expression using siRNA (Figure 6A) and explored its biological roles through loss-of-function assays.

To characterize the functional significance of Fas in lung cancer cells, we performed cell proliferation and apoptosis analysis assays after knocking down Fas. RNAi-mediated suppression of Fas for 24, 48, 72, and 96 h reduced cell viability and growth in a time-dependent manner (Figure 6B). To explore Fas regulation of lung cancer cell invasion and migration, we performed transwell invasion and migration assays. Fas knockdown decreased cell invasion and migration by approximately 51% and 42%, respectively, compared with the cells transfected with control siRNA (Figure 6C). Fas inhibition decreased the percentage of dead and apoptotic cells compared with the cells transfected with control siRNA (Figure 6D). These results indicate that silencing Fas reduces apoptosis and implicates Fas/FasL as mediators of PGL-induced apoptosis.

In this study, we demonstrated that Fas knockdown repressed cell growth, invasion, and migration and reduced apoptosis in lung cancer cells (Figure 6) and that PGL treatment induced apoptosis (Figure 4) and activated the expression of Fas/FasL (Figure 5). These data indicate that Fas is an important regulator of cell proliferation and apoptosis and that Fas/FasL signaling is essential for both cancer cell proliferation and resistance to anticancer drugs. These data provide the first evidence that PGL induces apoptosis by enhancing Fas and FasL expression, highlighting Fas as a potential target to enhance the effect of anticancer drugs.

## 3. Conclusions

In this study, the characteristics and antitumor activities of PGL were investigated. PGL is a neutral polysaccharide with a linear structure of repeated disaccharide agarobiose units consisting of 3,6-anhydro-l-galactose and d-galactose. Three main fractions of PGL, P-1, P-2, and P-3, were obtained with yields of 12.61%, 69.26%, and 18.70%, respectively. The average MWs of PGL, P-1, P-2, and P-3 were 123.06, 14.29, 64.78, and 57.02 kDa, respectively. PGL significantly inhibited cell proliferation, altered cell morphology, and induced cell apoptosis. Furthermore, we demonstrated that PGL inhibited proliferation by inducing apoptosis mediated by the Fas/FasL pathway in cancer cells. It is important to study the potential antitumor applications of natural polysaccharides, although the focus in polysaccharide research is gradually shifting to the prevention of and clinical intervention in neoplastic disease. Further studies should focus on the structural characteristics of polysaccharides, such as glyosidic linkages, the structure-activity relationship and the potential of the Fas/FasL pathway as a target to enhance the effects of antitumor drugs.

## 4. Materials and Methods

### 4.1. Materials

*Gp. lemaneiformis* 911 was collected from the coast of Wenzhou, Zhejiang Province in China (27°52′ N, 120°36′ E) in October 2014. *Gp. lemaneiformis* was cleaned of epiphytes, washed several times with distilled water, air-dried, ground in a mill to a fine powder, and vacuum frozen. The human gastric cancer cell line MKN28, the non-small cell lung cancer (NSCLC) cell line A549, and the mouse melanoma cell line B16 were purchased from the Chinese Academy of Sciences Committee Type Culture Collection Cell Bank (Shanghai, China).

### 4.2. Characterization of Polysaccharides from Gp. lemaneiformis

#### 4.2.1. Extraction of Crude Polysaccharides

Polysaccharides were extracted from *Gp. lemaneiformis* and purified according to our previous study [13]. Briefly, powdered *Gp. lemaneiformis* was extracted with a 90-fold volume of distilled water for 4 h at 80 °C. After centrifugation to remove residues, the supernatant was concentrated in a rotary vacuum evaporator. The concentrated solution was precipitated and re-dissolved in warm water. The dissociated proteins were removed by the Sevage method. The supernatant containing the polysaccharides was dialyzed using a dialysis bag with a pore diameter of 3500 D in distilled water for 48 h and vacuum freeze-dried.

#### 4.2.2. Isolation and Purification of Polysaccharides

Crude polysaccharides were purified sequentially by DEAE-A25 and Sephadex G-100 column chromatography (Sigma-Aldrich, St. Louis, MO, USA) according to previously described methods [21]. The polysaccharides were re-dissolved and fractioned using 30% and 70% ethanol solutions successively. Subsequently, the fractions were separated with a DEAE-Sephadex A-25 ion exchange column (30 cm × 3 cm) (Sigma-Aldrich, St. Louis, MO, USA), which was eluted stepwise at a flow rate of 0.5 mL/min with distilled water and 3 M NaCl. One hundred tubes with 5 mL per tube were collected, and the fractions of the yield were concentrated and combined using the anthrone-sulfuric acid method and describe the elution profile. Furthermore, the polysaccharides were eluted using distilled water and a step-wise gradient of NaCl solutions (0.3 and 1.2 M) at a flow rate of 0.5 mL/min, and 5 mL fractions from each tube were collected by an automatic fraction collector. Next, the polysaccharide fractions P-1, P-2, and P-3 were concentrated, dialyzed against distilled water, and further purified through a Sephadex G-100 column (30 cm × 3 cm) (Sigma-Aldrich, St. Louis, MO, USA) equilibrated with distilled water. Furthermore, the protein and nucleic acid contents in the polysaccharide fractions were assessed using ultraviolet spectrometry (180–640 nm wavelengths). Each fraction had only one main peak, and the main peaks were collected, dialyzed, desalted, concentrated, and lyophilized for use in subsequent assays [22].

#### 4.2.3. Determination of Molecular Weight

The MW of the polysaccharides was determined by PAGE gel chromatography [23]. The column was filled with Sephadex G100 gel and equilibrated with 0.05 M NaCl solution. Blue Dextran 2000 (1 mg) and standard Dextran (1 mg, molecular weight: 10,000, 20,000, 40,000, 70,000 Da) were dissolved in 1 mL distilled water and loaded onto the column, which was eluted with 0.05 M NaCl solution. The eluent was collected, and the amount of Blue Dextran was measured using the phenol–sulfuric acid method [24]. A standard curve was drawn according to the following logarithm equation: *K*_av_ = (*V*_e_ − *V*_0_)/(*V*_t_ − *V*_0_), in which *K*_av_ was the logarithm of the molecular weight, and *V*_e_, *V*_0_, and *V*_t_ were the elution volumes of standard Dextran, Blue Dextran 2000, and water, respectively. The elution volume of each polysaccharide sample (*V*_S_) was determined and used to calculate the average molecular weight according to the standard curve.

#### 4.2.4. Chemical Properties of Monosaccharides

The content of the polysaccharide fractions was determined using the phenol–sulfuric acid method with d-galactose as the standard. The protein content of the samples was measured according to the methods described in a previous study [25]. The polysaccharide sulfate radical content was determined with the barium sulfate turbidimetric method. In addition, the amount of 3,6-anhydro-l-galactose in the polysaccharide was quantified according to the resorcinol colorimetric method [26]. The monosaccharide constituents of the PGL fractions were characterized by GC-MS using an Agilent 7890A-5975C GC-MS (Agilent Technologies, Wilmington, DE, USA) with a DB-1 column (15 m × 0.2 mm, 0.33 μM, J&W Scientific, Folsom, CA, USA). The program used was as follows: the initial temperature of the column was 100 °C, was increased to 280 °C at a rate of 10 °C/min, and maintained for 15 min with an injection temperature of 280 °C. The temperature of the mass spectrometer ion source was 230 °C, and 1 μL of the sample was injected into the column with a split ratio of 10:1 [25]. Galactose (Gal), glucose (Glu), mannose (Man), xylose (Xyl), arabinose (Ara), rhamnose (Rha), and fucose (Fuc) were chosen as the standard monosaccharides. Calculation of the molar ratio of the monosaccharides was based on the peak area of the monosaccharides.

### 4.3. Assays for Antitumor Properties

#### 4.3.1. Cell Culture and PGL Treatment

Three cancer cell lines (MKN28, A549 and B16) were cultured in RPMI-1640 medium (Invitrogen, Carlsbad, CA, USA) supplemented with 10% fetal bovine serum (FBS) (Gibco BRL, Grand Island, NY, USA), 100 units/mL penicillin, and 100 µg/mL streptomycin at 37 °C in a humidified incubator containing 5% CO_2_. For the PGL treatment and antitumor analysis, cells were seeded into a 6-well culture plate at a density of 5 × 10^5^ cells/well and treated with serial concentrations of PGL (0, 5, 10, 20, 30, 40, 50, 60, 80, and 100 µg/mL) in a humidified atmosphere with 5% CO_2_ for 24, 48, and 72 h.

#### 4.3.2. Cell Viability Analysis

To investigate the effect of PGL on cancer cell viability, three cancer cell lines treated with different concentrations of PGL (0, 5, 10, 20, 30, 40, 50, 60, 80, and 100 µg/mL) were evaluated with a cell counting kit-8 (CCK-8). Briefly, 2 × 10^3^ cells were seeded in each well of a 96-well plate with 200 µL growth medium. After an overnight incubation, the cells were incubated with different concentrations of PGL in a humidified atmosphere with 5% CO_2_ for 24, 48, and 72 h. After the incubation, the media was replaced with 100 µL of fresh medium with a 10 µL CCK-8 working solution (Dojindo Molecular Technologies, Tokyo, Japan). After incubation at 37 °C for 3 h, the plate was read at a wavelength of 450 nm using a spectrophotometric plate reader (Bio-Tek Instruments, Inc., Winooski, VT, USA). The absorbance value of the control group (0 µg/mL) was considered to be 100%. Five independent experiments were used to collect the results.

#### 4.3.3. Observation of Cell Morphology Changes

To evaluate the effects of PGL (0 and 60 µg/mL) on cell morphology and growth, 1 × 10^5^ cells/well were seeded in 6-well plates and exposed to the half maximal inhibitory (IC_50_) concentration of PGL (as determined by the CCK-8 assay) for 48 and 72 h. The morphology changes of A549, MKN28, and B16 cells were observed using a phase-contrast microscope (Olympus, America Inc., Melville, NY, USA).

#### 4.3.4. Apoptosis Analysis

Apoptosis was determined with annexin V-FITC/PI staining using flow cytometry. A549, MKN28, and B16 cells were pretreated with different dilutions of PGL (0 and 60 µg/mL) for 48 and 72 h. Next, 1 × 10^6^ cells were collected, centrifuged, and washed twice with phosphate buffered saline (PBS). Then, 300 μL binding buffer was added to each tube to re-suspend the cells. The cells were incubated with 5 μL annexin V-FITC and 5 μL PI for 15 min at room temperature in the dark before flow cytometry analysis.

#### 4.3.5. Fas/FasL Expression Analysis

Total RNA was extracted with TRIzol reagent (Invitrogen Corp., Carlsbad, CA, USA). The RNA yield and quality were determined with a NanoDrop 2000 Spectrophotometer. The real-time PCR primers used for each gene were as follows: Fas (Accession number: NM_152872), 5′-TCTGGTTCTTACGTCTGTTGC-3′ (forward), 5′-CTGTGCAGTCCCTAGCTTTCC-3′ (reverse); FasL (Accession number: NM_000639), 5′-AACTCAAGGTCCATGCCTCTG-3′ (forward), 5′-GGTGAGTTGAGGAGCTACAGACA-3′ (reverse). For cDNA synthesis, total RNA was reverse transcribed using a Toyobo RT reagent Kit (Perfect Real Time) according to the manufacturer’s instructions. Quantitative real-time RT-PCR (RT-qPCR) was performed using SYBR Premix Ex Taq on an MX3000 instrument. The PCR amplification program consisted of initial polymerase activation at 95 °C for 10 min, followed by 40 cycles at 95 °C for 5 s, 60 °C for 30 s, and 72 °C for 30 s for the genes of interest. The results were normalized to the housekeeping gene β-actin. The relative level of mRNA was calculated as 2^−ΔΔCt^. 

#### 4.3.6. Western Blot Analysis

We investigated whether Fas protein levels changed in PGL-treated cancer. The three cancer cell lines were treated with PGL (0 and 60 µg/mL) for 24, 48, and 72 h, and the Fas protein levels were examined by western blotting. Western blotting was performed according to the standard protocol described in our previous study [27]. The proteins were extracted from A549, MKN28, and B16 cells, and approximately 50 µg of protein sample were separated by SDS-PAGE (10% polyacrylamide gels) and probed with a primary antibody against Fas (1:550, Sangong Biotech, Shanghai, China). Glyceraldehyde-3-phosphate dehydrogenase (GAPDH) (1:2000, Cell Signaling Technology, Beverly, MA, USA) was used as an internal control. Protein band intensities were quantified by densitometry using an Odyssey infrared imaging system ((Li-Cor, Lincoln, NE, USA).

#### 4.3.7. siRNA and Transfection

The small interfering RNAs (siRNA) targeted against human Fas (siFas, 5′-GUGCAAGUGCAAACCAGACTT-3′) and negative control siRNA (siControl, 5′-CCCCUUUUAAAAGGGGCC-3′) were purchased from Invitrogen. A549 cells were transfected with siFas or siControl to a final concentration of 50 pmol/mL using Lipofectamine RNAi Max (Invitrogen) according to the manufacturer’s protocol. After transfection of the siRNAs for 24–96 h, viable cells were quantified using CCK-8, apoptosis analysis was conducted with annexin V-FITC/PI staining using flow cytometry, and knockdown was assessed by RT-qPCR and western blotting from a parallel transfection.

#### 4.3.8. Cell Invasion and Migration Assays

Invasion and migration assays were performed as described previously [28]. Briefly, 8 μm pore transwell filters (BD Biosciences, San Jose, CA, USA) were used for the invasion assay, and transwell membranes were coated with Matrigel™ Basement Membrane (356,234, BD Biosciences, San Jose, CA, USA). Transfected cells were re-seeded onto the upper precoated chamber (2 × 10^4^ cells per well) in 100 μL serum-free medium. The lower wells of the chamber contained 0.6 mL 15% FBS-supplemented medium. The cells were incubated for an additional 48 h at 37 °C in 5% CO_2_. For the cell migration assay, transfected cells were re-seeded onto the upper chamber in 100 μL serum-free medium (5 × 10^4^ cells per well). Migration-inducing medium (with 10% FBS) was added to the lower chambers of the transwells, and the cells were incubated for an additional 24 h at 37 °C in 5% CO_2_.

### 4.4. Statistical Analyses

All of the experiments were performed in triplicate and presented as the mean ± standard deviations (SD). The data were analyzed using SPSS^®^ (version 18.0; SPSS Inc., Chicago, IL, USA). Differences in antitumor activities were analyzed using one-way analysis of variance (ANOVA). Correlations between the data obtained were calculated using Pearson’s correlation coefficient. *p*-values < 0.05 were considered significant.

## Figures and Tables

**Table 1:** Chemical properties and molecular weights of *Gracilariopsis lemaneiformis* (PGL) and its main fractions.

Samples	Crude PGL	P-1	P-2	P-3
3,6-anhydro-l-galactose (%)	41.20 ± 0.09	58.30 ± 0.04	47.60 ± 0.06	38.61 ± 0.05
d-galactose (%)	57.38 ± 0.04	29.53 ± 0.03	51.27 ± 0.05	59.76 ± 0.02
Sulfate ester (%)	9.24 ± 0.01	8.15 ± 0.02	9.16 ± 0.03	8.14 ± 0.02
Protein contents (%)	19.49 ± 0.08	0.35 ± 0.03	0.26 ± 0.02	0.42 ± 0.03
Molecular weight (kDa)	123.06	14.29	64.78	57.02
